# Clinician perspectives on policy approaches to genetic risk disclosure in families

**DOI:** 10.1007/s10689-024-00375-2

**Published:** 2024-03-28

**Authors:** Amicia Phillips, Danya F. Vears, Ine Van Hoyweghen, Pascal Borry

**Affiliations:** 1 Centre for Biomedical Ethics and Law, Department of Public Health and Primary Care, Leuven, Belgium; 2 Biomedical Ethics Research Group, Murdoch Children’s Research Institute, Parkville, Australia; 3 Melbourne Law School, University of Melbourne, Parkville, Australia; 4 Life Sciences and Society Lab, Center for Sociological Research, Leuven, Belgium

**Keywords:** Qualitative research, Genomics, Genetics, Policy, Ethics

## Abstract

Genomic sequencing has emerged as a powerful tool with significant implications for patients and their relatives, however, empirical evidence suggests that effective dissemination of risk information within families remains a challenge. Policy responses to address this issue vary across countries, with Belgium notably lacking specific regulations governing nondisclosure of genetic risk. In this study, we conducted semi-structured interviews with clinicians from Belgian clinical genetics centers to gain insight into their perspectives on policy approaches to the disclosure of genetic risk within families. Using real-world examples of legislation and court rulings from France, Australia, and the UK, we explored clinician viewpoints on the roles and responsibilities of both patients and clinicians in the family communication process. Clinicians expressed confusion regarding what was legally permissible regarding contacting at-risk relatives. While there was a consensus among participants that patients have a responsibility to inform their at-risk relatives, participants were hesitant to support the legal enforcement of this duty. Clinicians mostly recognized some responsibility to at-risk relatives, but the extent of this responsibility was a subject of division. Our findings highlight the need for a comprehensive policy that clarifies the roles and responsibilities of clinicians and patients to inform at-risk relatives. Furthermore, the study underscores the practical challenges clinicians face in supporting patients through the complex process of family communication, suggesting a need for additional resources and the exploration of alternative approaches to communication.

## Introduction

Findings from genomic sequencing can have important implications for patients and their relatives. When a genetic risk is present in a family, disseminating that information to all family members who are at risk in some cases can play a vital role in connecting them with preventive and treatment options. Furthermore, information about genetic risk can enable more informed reproductive decision making, and possibly prevent passing on the condition.

Although disseminating genetic risk information within families can be beneficial, initiating conversations about genetic risk can be very challenging. Conversations about genetic conditions can be distressing for patients and relatives, particularly when relationships are already distant or strained. So, despite the fact that many patients have the intention to inform their at-risk relatives, empirical research shows that in practice this communication does not always occur [1–4].

The responsibility to inform at-risk relatives falls predominantly on the patient’s shoulders [5]. While clinicians can support patients by offering a ‘family letter’ informing the recipients about their genetic risk and testing options, usually it is still the patient that is responsible for distributing these letters. In some cases, the clinician may distribute it on their patient’s behalf, with the consent of the patient. Genetics centers also commonly provide counseling and psychological support services to patients who need further help with informing relatives.

At a policy level, several approaches have been taken to address the issue of nondisclosure of genetic risk. For example, in France, legislation imposes a legal duty on patients to inform relatives, either themselves or via their clinician, about genetic risks with serious clinical or reproductive implications [6, 7]. Contrastingly, other countries focus their policies not on patients but instead on clinicians. Australia, for instance, has legislation that creates an exception to the privacy obligation that clinicians owe patients, thus granting clinicians the discretion (rather than obligation) to breach patient confidentiality to inform at-risk family members in cases where disclosure would be necessary to lessen or prevent a serious threat to genetic relatives [8–10]. It should be noted however that this legislation does not apply uniformly across all Australian states and between the public and private healthcare contexts. The UK has taken yet another approach to familial disclosure. In a recent judicial ruling, the court determined that clinicians have a duty to balance the interests of at-risks relatives (when a proximate relationship exists) who could benefit from being informed of their genetic risk with the patient’s interest in maintaining confidentiality [11]. This means that in the UK, there could be cases where clinicians are legally obligated to inform at-risk relatives. The cases in which these policies are applicable remains not entirely clear as there is no explicit list of conditions for which disclosure without patient consent is permissible, but rather each case is judged based on two main criteria: severity and actionability, although notably there is no consensus on the boundaries of both of these terms [5, 12–14].

Many other countries lack specific regulation adjudicating what can be done in cases of nondisclosure of genetic risk [12, 15]. This is the case in Belgium where there is no specific law or professional guideline regulating this issue [5, 12]. While existing legal frameworks might support any number of policy approaches, the lack of application thus far in the genetics context means that the interpretation of legal possibilities and responsibilities regarding this issue of nondisclosure remains unclear. In the likely case that clinicians encounter a case of nondisclosure, this lack of clear guidance and legislation may result in confusion and conflict over the best way to handle this ethically complex situation. In practice, due to the lack of clear regulation on family communication and cases of nondisclosure in Belgium, this means that patient led disclosure remains the norm and in cases of nondisclosure nothing can be done to inform at-risk relatives.

Although empirical research investigating clinicians’ views and experiences with existing laws has been conducted in several countries [6, 7, 16], no study has asked clinicians to compare different policy approaches. Furthermore, no study on the subject has been conducted in Belgium where there is a clear need for further consideration of the current policy gap. To address these gaps, we interviewed clinicians from Belgian clinical genetics centers to explore their views on various policy approaches to disclosure of genetic risk within families, including their perspectives on the roles and responsibilities of patients and clinicians in the process of family communication. To gauge clinicians’ perspectives, we used real world examples of the legislation and court rulings in France, Australia, and the UK.

## Methods

Participants were recruited via email invitation from all Belgian centers for human genetics (across the three regions of Flanders, Brussels, and Wallonia) using purposive sampling whereby we sought participants from different roles (clinical geneticists, genetic counselors, and psychologists) and with varying levels of experience. There was a participation rate of 56%, and recruitment continued until data saturation was reached.

We conducted semi-structured interviews of approximately one hour. The interview guide included open-ended questions regarding the experiences and perspectives of clinicians related to family communication, cases of nondisclosure, and policy approaches to disclosure. More specifically, the interview guide included themes touching upon the ethical and legal duties of patients and clinicians towards at-risk relatives, and further prompted participants by providing several examples of policy approaches, namely those found in France, Australia, and the UK. The interview guide was about genetic risk in general, rather than being focused on any particular condition, thus allowing participants to give examples and reflect based on their specializations. The interviews were conducted online (due to COVID restrictions) using Skype and Microsoft Teams (by AP) between May 2020 and January 2021. The interviews were voice recorded and then transcribed verbatim (YP and AP). The transcripts from the interviews were analyzed using inductive content analysis [17], whereby codes were derived from the data, rather than predetermined. Broad categories of codes were assigned in the first round of coding and then further developed into subcategories and fine-grained codes. These codes were then synthesized and refined through an iterative process. Coding was done using the software NVIVO by one researcher (AP) and then checked by a second senior researcher (DV). Discrepancies in quote selection and coding were discussed by the authors. The data analyzed for this study that focuses on clinician perspectives on policy approaches to nondisclosure was collected as part of a larger investigation that also assessed how clinicians addressed familial implications before and after testing, as well as clinician experiences with nondisclosure.

The ethics approval for this interview study was granted by the Leuven Social and Societal Ethics Committee, dossier no. G-2019 04 1625.

## Results

We conducted 19 interviews with clinicians from Belgian genetics centers to explore their views on various policies addressing disclosure of genetic information to at-risk relatives. Our results first give an overview of participant characteristics, and then explain clinician perspectives on the current regulatory context before then presenting their arguments for and against policy approaches to disclosure. More specifically the results cover perspectives on the default of patient led disclosure, followed by a presentation of attitudes regarding the roles and responsibilities of clinicians.

### Participant characteristics

A total of 19 clinicians participated, including fourteen (74%) clinical geneticists, four genetic counselors (21%), and one psychologist (5%). Clinicians were spread between the three regions of Belgium with fourteen from Flanders (74%), three from Brussels (16%), and two from Wallonia (10%). Participants had varied levels of experience, ranging from 1 to 35 years of experience, with a mean of 16 years of experience. Thirteen clinicians (68%) were women and six were men (32%). The rate of positive responses was 56%, with 34 clinicians being contacted. Most did not provide a reason for declining to be interviewed, although a couple from French speaking centers cited the language barrier due to interviews being conducted in English.

### Current situation: ambiguity of the Belgian regulatory and policy framework

Participants experienced confusion over what was currently permissible under the Belgian law. Several participants were uncertain whether in Belgium there were guidelines or legislation on family disclosure of genetic information:

“I don’t think that [there are] guidelines here or rules in Belgium, or at least I’m not aware of it, but I’m sure that there are some guidelines from the European Society [of Human Genetics] or the American Society [of Human Genetics] about this, but if you ask me ‘have you read these guidelines recently’ the answer is ‘no’.” (P1, clinical geneticist).

Other participants misunderstood what is currently allowed under Belgian law when cases of nondisclosure occur. While participants generally understood that clinicians were not allowed to directly contact at-risk relatives, several participants thought that it was legally permissible to contact the at-risk relative’s or partner’s clinician as an alternative means of informing relatives:

“I never did it, but I think we can do it. If the patient asks you to contact or to inform, you mostly give [them] a letter and they have to do it themselves. If they are not capable of doing so, I think as a geneticist you can contact a family practitioner and inform them that in the family of that person there is somebody with that disease, if [the family practitioner] is able to give that information anonymously, eh? But I never had to do it. So, I think if really necessary it’s an option.” (P2, clinical geneticist).

One participant gave an example of a time that they had intervened, not because a relative was at-risk, but rather the partner of the patient was at-risk of having an affected child. The participant believed that communication between clinicians was not a violation of patient confidentiality due to the allowance for healthcare professionals to communicate medical information amongst themselves:

“So, I had a case of Huntington’s where I had to contact the GP because it was a patient who had Huntington’s and already had been diagnosed twenty years ago. But he was sexually very active and he got a lot of women pregnant…So I had to go all the way around to tell, it was not a GP in this case, it was a gynecologist, who was following the woman. I had to communicate that, just to state the risk, right? Of course, I was not permitted to talk to the woman myself.” (P3, clinical geneticist).

When asked about how to improve how cases of nondisclosure are handled in Belgium or in their practice, some participants felt that having clearer legal guidance would be helpful:

“We all know about HIV where you can tell the family. And then in every case we discuss like that there’s someone mentioned, “Isn’t it just like the infectious diseases and we can tell them?”, and then one other person says, “No, I don’t think we can.”, and then it stops. So, first of all, we need more information about what we can do and what we can’t. And then we need a clear law. Like in France or even in Australia. Even if I don’t think that’s the best solution, it’s a solution.” (P8, clinical geneticist).

One participant pointed out how having guidelines could help assuage the discomfort of the ethically challenging situation created by nondisclosure in the absence of a clear regulatory framework:

“I think it’s good to have guidelines because sometimes in some situations you need them to really feel comfortable in making a decision and in your conclusions. But of course, guidelines are guidelines. One situation is not the other one and it’s very difficult to make black and white guidelines. Guidelines are guidelines in the sense that it’s something you can use in case you have doubts.” (P1, clinical geneticist).

On the other hand, another participant questioned the added value of guidelines and legislation on nondisclosure:

“I think there is no law against it, and there is no law permitting you to do so, so we don’t need a law. …I guess we all have these guidelines, and nothing is really written down, but we know how to do it in practice. …Everybody knows that it’s important to discuss implications for family members with your patient. I’m not sure what guidelines will actually change. It’s more like a tick box.” (P6, clinical geneticist).

### Patient role in disclosure

All participants agreed that patients have an ethical duty to inform at-risk relatives when the condition in question was both serious and actionable. Generally, participants felt patients should be the primary informants on the basis of their relationship with the family members:

“It’s still the responsibility of the patient, because as a healthcare professional, you don’t know the familial situation. If we go around the patients to other family members, maybe we can do more damage than good in some cases. So, if the patient is obliged to distribute this information, anonymously or not, then I think in most cases, this will be the best solution.” (P15, genetic counselor).

One participant even stated how having the patient be the one to share information about genetic risk could be an act of caring that could help strengthen bonds:

“I think that as a health professional, we have the duty to support people in communication with their families, but they have to take the lead. In the end, that will work better. For inter-familial relations, I think it might be better if [disclosure is done] by the person themselves and not by a complete stranger, because at least it tells the family members that somebody in their family is taking care of them by giving them information and by leading them to a specialized health care professional.” (P5, clinical geneticist).

Many stated that it was already common when returning results of genetic testing to emphasize to patients the familial implications of the results and the patient’s responsibility to inform their at-risk relatives. In some genetics centers, this issue was already addressed prior to testing as part of the informed consent process, with some written consent forms clearly stating that patients have a moral duty to disclose information about genetic risk to relatives. The degree to which discussing familial implications and corresponding responsibilities with patients was feasible, particularly prior to testing, was dependent on the context and scope of the genetic testing being performed.

While there was consensus on the patient’s ethical duty to inform, participants differed on whether the patient’s moral duty to disclose should correspond to an additional legal duty. Some participants supported a policy that would add legal backing to the patient’s duty to inform:

“I think if people have to sign something, they really become aware of the importance of it. …I think that it should be clear that it’s at least a moral duty, and secondly, if you have to go that far, to say that they are legally responsible for it.” (P12, clinical geneticist).

Many of those who supported this policy also added that while the duty to communicate should mainly be that of the patient, clinicians had a duty to support patients by providing services such as counselling.

More commonly, support for an ethical duty to disclose did not correspond to support for a legal duty to disclose, and many participants questioned establishing a legal duty for patients:

“From my point of view, I don’t really see the need to legalize it. …especially for our situation, because I can tell you most of the time, we do see family members. …I don’t see the necessity to make it legal.” (P13, genetic counselor).

Some participants opposed establishing a legal obligation in principle because they did not think patients should be forced to do something against their will:

“Would be nice from a doctor’s perspective, but for my liberal view, I think it’s hard to say that you can really push people. …Because from a legal point of view, I don’t think you can force anyone to do something that he doesn’t really want to do. You can say the medical issues are higher than privacy issues, but patients might have another opinion about that.” (P11, clinical geneticist).

Another participant worried that forcing patients might be too onerous, particularly for patients who had to cope with their own treatment and health issues:

“I think it puts quite a burden on families, individual patients, right? …It makes it more stressful. I think it does more harm than it really helps people, so I would not put an obligation in the law. I think it’s more a moral ethical issue, rather than a legal issue.” (P6, clinical geneticist).

The main reason many participants were hesitant to support policies that would formalize this responsibility was due to their fear of the legal consequences:

“Having them sign a paper where they solemnly swear that they will inform their family members, and if they fail to do so, they can be prosecuted is so disruptive for patient-doctor relationship. …The problem is in these families where it’s already difficult, signing a paper will not help. They won’t communicate. They will say well, [my relative] can sue me. And then you create more problems than you actually solve.” (P6, clinical geneticist).

Some participants were also concerned that patients having a legal duty to disclose could impact their willingness to test:

“I support giving adults responsibility but I’m not in favor of the possible legal consequences, because that might be a very big barrier for people to do genetic testing. The result can be [that patients think] “I don’t want to communicate to the family so I’m not going to test.” Even without this legislation, the patient is responsible for this communication. …That’s why I think that if you make a law that forces the patient to communicate, it might just backfire.” (P18, clinical geneticist).

There was also the concern that this law could negatively impact the fiduciary nature of the doctor-patient relationship:

“If you could say “If you don’t do it, you will be penalized” and it would convince the patients, I don’t feel it would be very good for the confidence between the patients. So I don’t feel it would be the solution…” (P4, clinical geneticist).

Finally on a practical level, some participants questioned to what degree a legal duty for patients to inform relatives, if established, would even be enforceable:

“I think it’s very difficult to put that responsibility on the patient and to make it a legal obligation. It’s something that you cannot control. If you wanted to penalize individuals if they don’t do their duty, it’s difficult to know have they done it? Have they done enough? Were they clear enough? Or was it the relative who did not understand what testing was? So, I think it’s a very delicate matter.” (P14, clinical geneticist).

### Clinician-led disclosure

Participants widely agreed that they had some responsibility towards at-risk relatives. Several participants felt that by informing the patient of the importance of family communication, they had discharged their duty to the at-risk relatives. Some participants accepted that in some cases passing the responsibility on to patients might result in some at-risk relatives not being informed:

“And most of the times in the discussions, you can really work it out. It’s only very, very few cases that you really can’t come any further. I rest there because you can do your best, but sometimes it just stops. You can’t prevent everything.” (P11, clinical geneticist).

Some participants felt that their role in informing relatives should be determined by whether or not the patient consented to disclosure. The main argument given for supporting such a policy was based on the view that it is important that patients be the ones to decide, and that the role of clinicians is to respect that choice:

“If they refuse, even if you insist? I think you have to respect the [patient’s] decision, because insist and explain, but then if they don’t want [to inform relatives], I think you have to respect that. Even if I send a letter to a member of the family, it’s always because of consent of the patient. Just give me the address and I do the job, but I need the consent. …If the patient doesn’t consent, I think I have to respect their choice.” (P4, clinical geneticist).

Other participants felt conflicted about not informing at-risk relatives, because they felt that they owed more to at-risk relatives and wanted to do more to ensure disclosure. While generally participants agreed that patients should have the primary responsibility for informing their at-risk relatives, several participants supported clinician-led disclosure as a last resort when relatives would otherwise not be informed:

"First of all, we have to make clear to patients that it’s their duty. There might be a legal aspect to this that really stresses to the patient that it is their duty. Secondly, we have to help them because it’s not always easy to do. And then thirdly, when we are confronted with a situation where the information has not reached a certain level of distribution in the family, we should be able to breach confidentiality. …But then I’m not thinking in an active way, but more in a passive way, where we are not going to look for family members and trace them, but where when we see that [nondisclosure has occurred] we can breach confidentiality.” (P12, clinical geneticist).

One participant even supported the establishment of a duty for clinicians to disclose:

“At least there should be an obligation to communicate. …And so yes, I think so, especially when there are many implications for the care of this potential effect. And, of course, then it’s up to the family member to decide whether he will undergo a test or not, of course, everybody should be free but at least get the information. If they don’t want to know, that’s a possibility, but I think the information should be given.” (P17, clinical geneticist).

Most participants were skeptical of clinician led disclosure. Participants were particularly concerned about a policy approach that would enable clinicians to disclose without the consent of the patient, which many saw as a breach of confidentiality. Several participants voiced concerns regarding the impact of disclosing without consent on their relationships with patients:

“I don’t think we should. It’s really nasty in my opinion, if when you hear you have bad results, [you are told] “Okay, you don’t want to communicate it, I’m going to do it for you, even if you do not want to.” I think it may really hurt people and also it hurts your relationship with a patient.” (P18, clinical geneticist).

Participants were concerned about the additional responsibilities that clinician led communication would require.

“I believe that it is really the responsibility of the patient. I don’t think that if there would be guidelines that we were allowed to do so, I don’t know if we would do it, because do we have the time to do this to start contacting 10 family members? And then let’s say that you cannot reach two of them, but you still have to do it? …It would give us a responsibility that that is not ours. It will put a big, big load on us, emotionally and practically to do so.” (P16, psychologist).

Furthermore, several participants were worried about contacting relatives with whom they did not have a pre-existing relationship of care:

“I think if it depends a lot on the probability that you would do harm to a family or to a patient. If you don’t know family and if you don’t know how communication is within a family, it’s very difficult to evaluate as a healthcare worker who is outside of the family. …I think if you would be in close contact with family it would be easier to evaluate the potential harms or benefits, but if you communicate in an incorrect way within a family, it can lead to major conflicts.” (P14, clinical geneticist).

The main reason participants did not support a policy enabling clinician led disclosure, whether in cases where the patient had actively objected to disclosure or had passively failed to inform relatives, was due simply to practical concerns and limitations.

“We had a couple of cases in our department where we were really sure the patient wasn’t going to tell anyone. And most of the time, if they don’t tell us, we don’t have the contacts for the family. So yeah, then it’s not in our hands anymore, because we aren’t the FBI, we can’t.” (P8, clinical geneticist).

To help aid with the practical concerns, some clinicians supported the involvement of other health care professionals such as genetic counsellors and general practitioners.

“We should reorganize our centers because it’s a lot of work to [inform relatives] by ourselves. Maybe the genetic counselors could also help to contact people and to explain, but the medical doctors, we do not have enough [resources] to be able to do it. And it would also be dangerous because then we could be at fault if we don’t call all the family members. I think it’s a lot. It’s a huge undertaking. It should be done by genetic counselors because the medical doctors don’t have time to do it.” (P4, clinical geneticist).

Several participants drew parallels between the infectious disease and genetics context, and wondered whether a public health approach could be adopted for at least some genetic conditions. One participant expressed how having someone other than the treating clinician being the one to inform relatives could be a good approach:

“I think it’s easier if there is some third person, like a registry or someone to inform the family, not the doctor themself. But it is better [that patients] are informed in advance, I think… I think it’s [a] good [approach] for really common diseases. …I think it’s good because there’s a whole system. It’s there, and then the patients know that if they get tested, their family will be informed. It’s not a question of “Will somebody do it, who will do it?”, but it’s just, it is like that, it’s the system.” (P8, clinical geneticist).

A participant pointed out though that the implementation of such an approach requires certain healthcare structures be in place and how that context may influence the acceptability of a public health approach to informing at-risk relatives:

“Denmark is a great country from epidemiological point of view, because all those [health care] systems are connected over there… [Generally in Belgium] you never know if [genetic risk] is really communicated. Because in [Walloon town], we live in a rather isolated region…more or less we know who is seeing who and whether family members have been reached. But something like [the Danish public health approach] would be great. On the other hand, I think that the Danish are so used to their personnel doing their administration, and it’s not like it’s here in Belgium. So, I think most people would be shocked by the idea.” (P10, clinical geneticist).

## Discussion

This study asked clinicians to compare different policy approaches to disclosure of genetic risk to at-risk relatives, which is of particular relevance in Belgium where there is currently no specific policy addressing nondisclosure of genetic risk. One of the overarching questions of this research was on the added value, if any, of developing a policy addressing cases of nondisclosure in Belgium. A few clinicians that we interviewed were skeptical of the need to create guidelines and legislation for an issue they felt was common sense. However, many participants had questions over what was possible under the current legislation. Concerningly, several participants misunderstood what was currently allowed, with multiple participants discussing the possibility of contacting a family member’s clinician to inform them that a genetic condition had been identified in the family when the patient was unwilling to inform those at-risk. While professional secrecy between clinicians is legally recognized by Belgian legislation, case law, and legal literature [12], the application of shared professional secrecy in this context is problematic. This is because the two clinicians who would be involved in the discussion of confidential patient information (i.e., the patient’s clinical geneticist and family member’s general practitioner or gynecologist) are not involved in the care of the same person. Furthermore, while in some cases professional secrecy allows information to be shared without consent, that is only possible when it is in the interest of the patient themselves, so would not extend to the interests of family members [12]. When participants encountered cases of nondisclosure, it was clear that many participants felt conflicted and struggled to navigate the best course of action. For this reason, many participants supported further clarification or development of policies regarding nondisclosure of genetic risk.

Despite the support for the development of a policy addressing nondisclosure, participant perspectives differed regarding what policy approach they thought best balanced the interests of patients, at-risk relatives, and clinicians. It should be noted that participants came into the interviews with varying degrees of pre-existing knowledge regarding the various policy approaches to disclosure, meaning for some this was a topic that they had already contemplated in-depth and had clear opinions on while for others the interview was one of the first times they heard of the different policies and gave them consideration.

Our results found a general consensus that patients had an ethical duty to disclose, but when it came to translating this to a legal duty participants raised many questions and concerns regarding implementation and enforcement. On the one hand, participants were unsure how such a policy could even be enforced, but on the other hand they also feared that the enforcement of the duty to disclose could lead to legal consequences for patients. Notably, the patient’s legal duty to inform at-risk relatives has already been codified in France where patients must either inform their at-risk family members themselves or provide the contact information of their family members so that clinicians can contact their at-risk relatives. Patients who fail to inform their relatives cannot be criminally sanctioned but may be held liable under civil law. While this is legally the case, empirical research conducted with French clinicians suggests a softer or more indirect effect of the law, whereby the legislation largely did not change clinical practices, but instead emphasized to clinicians and patients the importance of family communication [6, 7]. French clinicians reported that even with the law in place they cannot know whether patients actually informed their relatives, and furthermore even if they find out that the patient has not informed their relatives, there is nothing that they can do to enforce it without the patient’s consent and cooperation [6, 7].

Participants in our study feared that creating a legal duty for patients to inform their relatives could negatively impact the clinician-patient relationship and deter patients from receiving genetic testing in the first place. There is no research evidence yet to confirm these concerns [6, 7, 18], so it still is uncertain to what degree this would be an issue. Our data showed that clinicians were hesitant to go against patient wishes, but empirical research conducted with patients does not reflect these same concerns and instead indicates most patients do not actively object to informing their relatives [7, 19]. It is thus unclear to what degree patients would feel their wishes had been violated in this context. Our findings suggest that the impact of the law on patients and their relationships with clinicians is likely to be dependent on to what degree the law would be enforced. More research is needed to understand to what degree patient perspectives align with the clinicians’ concerns.

Generally, participants did feel that they had an ethical responsibility towards at-risk relatives. Where they differed though is in how they interpreted the fulfilment of that responsibility. Several participants felt that by informing the patient of the importance of family communication, they had discharged their duty and done all that they could given the difficult situation. This interpretation of a responsibility towards relatives is consistent with the American policy approach, whereby the clinician’s duty to warn at-risk relatives is satisfied by informing patients and supporting them in disclosure [20]. Our data indicated that not all clinicians would be satisfied with this approach. Several participants felt they should do more for at-risk relatives, and thus experienced a tension between what they thought was the right thing to do and what appeared to be permitted under current interpretation of the Belgian law. These participants presented supporting arguments for a policy that would enable clinicians to inform at-risk relatives, although this was seen as a last resort when patients were unable or unwilling to communicate. Alternatives to patient-led disclosure have been gaining traction, with countries such as the Netherlands, Switzerland, Sweden, Denmark, and Finland investigating the acceptability of different forms of direct contact in recent years [21–24]. While there has been a lot of skepticism towards moving away from a purely patient-led approach to disclosure, an increasing amount of literature shows that patients and the public may not be averse to disclosure led by clinicians or healthcare services. Yet, as attitudes towards direct contact may be impacted by cultural factors, findings from previous empirical studies may not be generalizable to all contexts [22]; more research is necessary to assess the acceptability of this policy approach across different countries and cultures.

Participants questioned whether they were best situated to inform at-risk relatives if a direct contact (clinician mediated) approach was taken. The main reason was practical and concerned with feasibility: they worried about the amount of time and resources required, which was consistent with research on clinician perspectives conducted in other countries [7, 16, 25]. For this reason, several participants stated that while they would like the ability to inform at-risk relatives if they happened to become aware of a case, they would not take further measures to seek out family members. Additional reasons that participants questioned a direct contact approach was due to their lack of relationship with relatives and concerns for the impact on their relationship with patients. Empirical research with patients indicates that patients may not have the same concerns identified by clinicians. For instance, a qualitative study conducted with patients [25] indicated that they can distinguish between personal and familial information, meaning they might not perceive disclosure as necessarily being a breach of their confidentiality, as clinicians in our study were concerned about. An additional counterargument to the concerns voiced by our participants, is that while informing relatives could have unintended consequences, *not* informing relatives “could equally be worse for trust in the health service, because relatives develop a preventable cancer, where [clinicians] had known about the risk but had chosen not to act, might question their practices” [25]. Several participants wondered whether other health care professionals, such as the family member’s general practitioner or a public health service, might be better situated to communicate the discovery of a genetic risk in the family. In particular, general practitioners’ involvement in the dissemination has been under explored in the literature [26] and warrants further investigation.

## Conclusion

This study is the presents and analyzes clinician perspectives on policy approaches to disclosure of genetic risk. This work is of particular importance in a country like Belgium which currently lacks a specific policy to address the disclosure of genetic information to relatives. While from our relatively small sample we did not observe significant variations in perspectives based on participant characteristics, the development of future investigations, such as quantitative research could be beneficial in this regard. Our research indicates that clinicians experience confusion and conflict regarding decision making in cases of nondisclosure, particularly given the ambiguity of the Belgian legal situation. While there was a variety of perspectives expressed, most clinicians did support the development of a clear policy to clarify their role and responsibilities in the process of family communication. Most clinicians supported the idea that patients have a duty to communicate but were hesitant to endorse the legal enforcement of such a duty. While most clinicians felt that they had some responsibilities towards at-risk relatives, they were divided on to what lengths they had to take to inform relatives. Many arguments presented were practical in nature, indicating that further support and resources and needed to support clinicians, and in turn patients, through the challenges of family communication. Our research indicated there is some support for alternatives to patient led communication and to developing methods for better supporting patients in the process of informing their at-risk relatives, but further research is needed to explore these approaches.
