# Correction: Clinician perspectives on policy approaches to genetic risk disclosure in families

**DOI:** 10.1007/s10689-024-00409-9

**Published:** 2024-07-09

**Authors:** Amicia Phillips, Danya F. Vears, Ine Van Hoyweghen, Pascal Borry

**Affiliations:** 1 Centre for Biomedical Ethics and Law, Department of Public Health and Primary Care, Leuven, Belgium; 2 Biomedical Ethics Research Group, Murdoch Children’s Research Institute,, Parkville, Australia; 3 Melbourne Law School, University of Melbourne, Parkville, Australia; 4 Life Sciences and Society Lab, Center for Sociological Research, Leuven, Belgium

**Correction: Familial Cancer**

10.1007/s10689-024-00375-2

The article “Clinician perspectives on policy approaches to genetic risk disclosure in families”, written by Amicia Phillips, Danya F. Vears, Ine Van Hoyweghen & Pascal Borry, was originally published Online First without Open Access. After publication in volume 23, issue 2, page 71–80 the author decided to opt for Open Choice and to make the article an Open Access publication. Therefore, the copyright of the article has been changed to © The Author(s) 2024 and the article is forthwith distributed under the terms of the Creative Commons Attribution 4.0 International License, [ continue with the appropriate plain license text given in Creative Commons and OGL Licence Text for Open Access Content] [funding information]

The original article was updated.

